# Small changes, big gains: A curriculum-wide study of teaching practices and student learning in undergraduate biology

**DOI:** 10.1371/journal.pone.0220900

**Published:** 2019-08-28

**Authors:** Laura K. Weir, Megan K. Barker, Lisa M. McDonnell, Natalie G. Schimpf, Tamara M. Rodela, Patricia M. Schulte

**Affiliations:** Carl Wieman Science Education Initiative, University of British Columbia, Vancouver, British Columbia, Canada; Indiana University Bloomington, UNITED STATES

## Abstract

A growing body of evidence has shown that active learning has a considerable advantage over traditional lecture for student learning in undergraduate STEM classes, but there have been few large-scale studies to identify the specific types of activities that have the greatest impact on learning. We therefore undertook a large-scale, curriculum-wide study to investigate the effects of time spent on a variety of classroom activities on learning gains. We quantified classroom practices and related these to student learning, assessed using diagnostic tests written by over 3700 students, across 31 undergraduate biology classes at a research-intensive university in the Pacific Northwest. The most significant positive predictor of learning gains was the use of group work, supporting the findings of previous studies. Strikingly, we found that the addition of worksheets as an active learning tool for in-class group activities had the strongest impact on diagnostic test scores. This particular low-tech activity promotes student collaboration, develops problem solving skills, and can be used to inform the instructor about what students are struggling with, thus providing opportunities for valuable and timely feedback. Overall, our results indicate that group activities with low barriers to entry, such as worksheets, can result in significant learning gains in undergraduate science.

## Introduction

It is well-established that active, student-centered classrooms in undergraduate STEM education improve student outcomes compared to traditional lecture. These positive effects of active learning have been documented within individual courses; active approaches in high-enrollment introductory courses show improvements in student learning, engagement, attendance, attitudes, and retention in a course or program [1–6]. This pattern is remarkably consistent despite considerable variability in approaches to active learning and the magnitude of the impact of these approaches across courses. Freeman et al.’s [7] meta-analysis of over 200 published STEM studies indicates that active learning improved student performance and decreased failure rates irrespective of discipline, class size, course level, and instructor experience. However, the degree to which different active learning methods relate to variation in student success remains an open question. In this study, we explore the effectiveness of different active learning tools by examining how they relate to student success.

In practice, “active learning” in STEM education encompasses a wide variety of approaches that include collaborative learning, team-based learning, think-pair-share, and peer instruction [8]. While these techniques may include different tools (e.g., personal response systems such as i>Clickers, paper worksheets), most include a considerable amount of group work. When implementing activities from the literature, instructors often adapt in-class approaches to suit their own classrooms and teaching style. Thus, to understand the impact of varied classroom practices on student outcomes, it is essential to understand the variety of activities that occur in a typical lecture period in real classrooms.

With few exceptions [2,4], most of the STEM literature that focuses on the use of active learning approaches is based upon instructor self-reports, qualitative surveys, or indirect observations of active learning. These classroom measures are usually not generalizable and can be inaccurate [2,9–11]. Recent classroom observation tools such as the Classroom Observation Protocol for Undergraduate STEM (COPUS) [12] and the Practical Observation Rubric To Assess Active Learning (PORTAAL) [13] have been developed to provide a systematic and quantifiable estimate of the diversity of classroom practices and use of class time. These tools allow for a quantitative measure of the time spent on different activities in the classroom and objective comparisons across classes within or among courses. For higher-level comparisons among courses, data can be clustered to represent broad instructional styles across a continuum of approaches ranging from instructor-centred to student-centred [14].

Assessment of the success of different active learning approaches requires quantifiable and comparable measures not only of classroom activities, but of student learning as well. Multiple-choice conceptual inventory tests, or diagnostic instruments, are broadly used tools that allow for objective measurements of student thinking that are independent of course-specific quizzes or examinations [15–17]. Because of their informative power and ease of implementation, an abundance of rigorously evaluated conceptual inventory tests are readily available in the published literature (e.g., [15,16]). When combined with direct classroom observation, change in student performance on these inventories can provide robust evidence for the impact of various active learning practices.

In this work, we aim to characterize instructional styles in use across a range of biology courses at a large research institution, and to investigate the relationships between student learning and specific teaching practices without experimental manipulation of class activities. Student learning outcomes are measured by comparing pre- and post-course performance on conceptual inventory tests that align with the core concepts for each course. Given the large body of literature indicating that active learning enhances student performance, we predict that classes employing a student-centred approach will exhibit relatively higher student scores on concept inventory tests compared to classes that use traditional lecture. In addition, we examine the effect of different types of specific active learning practices on student performance. By accurately documenting the range of approaches used across classrooms, we can investigate which classroom activities contribute to improved student performance.

## Materials and methods

### Cohort

This study focused on the Biology program at the University of British Columbia, a large, research-intensive university in Vancouver, British Columbia, Canada. All instructors teaching biology courses without a laboratory component during Fall 2014 and Winter 2015 were contacted to take part in the study; a total of 31 class sections participated, with an average of 211 registered students per class. Class sizes ranged from numbers in the teens in fourth year courses to over 300 in first year courses. We chose to focus on courses without an integrated laboratory component so that we could assess student learning in primarily classroom environments. The 31 classes without a laboratory component that we studied here represent approximately 40% of all courses that are offered by the biology program in a given year. As incentive to participate, all instructors were offered the opportunity to see the aggregate data from their course (COPUS observations and student performance on concept inventories). The courses involved in the study were largely lower division (first- and second-year) courses; six of these courses were required for biology majors (out of a total of seven required courses). Many of these lower division courses were run as multiple different sections, each taught by a different instructor. Each instructor-unique course section was analyzed independently because each instructor offered a different approach to teaching. Students in the participating classes were asked for consent to use their data; only data from students who wrote both the pre- and post-test and gave consent were included in our analyses. No additional incentives were given to students. A breakdown of the courses and number of participants is shown in Table 1. This work was performed under approval from the UBC Behavioural Research Ethics Board, H14-02293.

**Table 1:** Description of cohorts used in this study.

Level	*n* unique courses	*n* unique sections	*n* matched students (% of total registered)	mean number of students per class
100	2	13	1431 (47%)	235
200	4	9	1773 (70%)	281
300	5	5	413 (47%)	175
400	4	4	48 (56%)	21
**Total**	**17**	**31**	**3728**	

The percentage of total students registered reflects the numbers in the courses that we surveyed, rather than all courses in the curriculum.

### Compiling conceptual tests

We quantified student learning as a change in score on a concept inventory test. Seventeen different conceptual inventory tests were administered, corresponding with 17 different courses in the study. These conceptual tests varied in length (6 to 23 questions) and were composed of a combination of questions that were either based on previously used test questions created by the researchers, sourced from validated inventories, or modified slightly from the validated questions. A complete breakdown of question sources and calculations of discrimination indices are available in S1 Table and S1 Fig. Tests were compiled collaboratively with instructors to match the central course content and learning objectives. Pre-tests were administered before any exposure to the content in that class, and post-tests were run at end of the semester (most during the last week of class). Researchers administered both the pre- and post-tests in person during class time. For each course, questions were presented at the start and end of the semester in one of two ways: 1) they appeared on lecture slides and students answered them on hard copy bubble answer sheets or voted on the answers with i>Clicker personal response systems; or 2) students were given a paper copy of the test and filled in their answers on a bubble sheet. The approach taken to deliver the questions and the method used to answer was consistent within each class for the pre- and post-tests. All matched scores for concept inventory tests are available in S1 File.

### COPUS observations

The COPUS protocol [12] was used to gather in-class observational data because it allows for live collection of quantitative data. A group of seven researchers, including six post-doctoral teaching fellows and an undergraduate student, conducted the classroom observations. All of the observers were trained to use COPUS prior to the study. Seven “practice” classes were attended and scored by more than one observer, and the intra-class correlation for the total number of each COPUS category was calculated for these observations using the ICC package in R [18]. This approach determines the degree to which values within a category are in agreement between observers; values for intra-class correlations vary between 0 and 1, and estimates greater than 0.75 are considered to be excellent inter-rater agreement [19]. The intra-class correlation for each class observed by more than one researcher ranged from 0.86 to 0.98, with a mean of 0.93. Data from the practice observations was not used in further analysis; raw data are included in S2 File.

Class observation data were collected for a “typical week” of the course, consistent with the approach used by Lund et al. [14]. This included approximately 150 minutes of class time that occurred in either three 50-minute classes or two 80-minute classes. The data were collected during weeks eight to ten of the 13-week semester. We avoided any irregular class sessions such as midterms. In total, 98 COPUS observations were made. Data from a particular section were averaged to obtain one value across observed sessions, such that each section of a particular course had only one set of values. This approach was used to reflect the time spent on different activities for courses in which classes on different days might have different structures (e.g., introduction of a topic on Tuesday, worksheets or other activities to promote understanding on Thursday). Raw and summarized COPUS data are available in S2 File. To assess the between-class variation within a course section, we examined pairwise correlation coefficients for the frequency of different class activities across the set of observations within a particular course. The average pairwise correlation between classes within the same section was generally very strong; in 24 of 31 sections observed, correlation coefficients were greater than 0.7, and 6 of the remaining 7 sections had correlation coefficients between 0.5 and 0.7, representing strong to moderate relationships. No sections had negative correlations between class periods. Thus, the data we used in subsequent analyses should accurately capture the total duration of different classroom activities.

### Classroom characterization

We first created broad categories of each of the 31 course sections using the methodology of Lund et al. [14]. These categories are ‘Mostly Lecture’, which encompasses both traditional and socratic lecturing, ‘Emergence of Group Work’, which includes practices ranging from limited to extensive peer instruction, and ‘Extensive Group Work’, which involves student-centered peer instruction and group work. COPUS code abbreviations used in this study, including definitions from Smith et al. [12], can be found in Table 2. Activities that occurred on average 5% of the time or less in the observed classes were removed from subsequent analyses. Instructor administration (I-Adm) was not a variable of interest for our study, and thus was also removed from our analyses. Following Lund et al. [14], we eliminated redundancy by including only the student component of any student-instructor pair of variables that were very tightly and significantly correlated (see S2 Table for full correlation matrix). This occurred for S-Q and IAnQ (r = 0.96, p<0.001), IPQ and SAnQ (r = 0.92, p <0.001), and S-CG and I-CQ (r = 0.95, p <0.001; see S2 Table for full correlation matrix. Student listening occurred relatively frequently (on average in 85% of two-minute time intervals), and was positively correlated with instructor lecturing (r = 0.76, p<0.001) and negatively correlated with group work (worksheets: r = -0.58, p <0.001, other group work: r = -0.53, p = 0.004) and instructors moving in groups (r = -0.77, p <0.001). Because we retained the other variables, we excluded student listening from our analyses to minimize spurious results. Thus, we retained the following five student codes for our analyses: S-CG, S-WG, S-OG, S-AnQ, and S-Q. Student group work variables (S-CG, S-WG, and S-OG) were re-coded to a ‘Student group work’ (S-GW) variable [14], to reflect the amount of time spent on group work, regardless of type, and to account for positive correlations among the three types of group work. For each time interval, we counted whether any group work occurred and included it only once to avoid double-counting group work. This approach reduced the number of student variables to three (S-GW, S-ANQ, and S-Q). The four instructor codes that were used in our analyses were I-Lec, I-RtW, I-FUp, and I-MG.

**Table 2:** Summary of COPUS observations for 31 undergraduate biology sections.

Classroom activities	abbreviation	mean ± standard deviation of percentage of class time	*n* sections in which activity occurred
**Student activity**			
Listening to instructor/taking notes, etc.	S-L	85.21 ± 12.41	31
Individual thinking/problem solving	S-Ind	4.17 ± 6.14	16
Discuss clicker question in groups of 2 or more	S-CG	14.07 ± 11.69	26
Working in groups on worksheet activity	S-WG	5.27 ± 8.69	10
Other assigned group activity	S-OG	15.34 ± 11.75	26
**Any type of student group work**	**S-GW**	33.20 ± 17.66	29
**Student answering a question**	**S-AnQ**	26.50 ± 12.41	30
**Student asks question**	**S-Q**	14.28 ± 9.42	30
Engaged in whole class discussion	SWC	1.45 ± 4.35	8
Making a prediction	S-Prd	0.04 ± 0.23	1
Presentation by student(s)	S-P	0.22 ± 1.20	1
Test or quiz	S-TQ	NA	0
Waiting	S-W	1.08 ± 1.88	12
Other	S-O	1.96 ± 2.70	16
**Instructor activity**			
**Lecturing**	**I-Lec**	53.47 ± 23.54	31
**Real-time writing on board, projector, etc.**	**I-RtW**	14.46 ± 15.80	22
**Follow-up/feedback to entire class**	**I-FUp**	36.67 ± 17.69	30
Posing non-clicker question to students (non-rhetorical)	I-PQ	31.10 ± 14.97	30
Asking a clicker question	I-CQ	17.52 ± 12.86	27
Answering student question with entire class listening	I-AnQ	15.21 ± 9.10	30
**Moving through class and guiding ongoing student work during active learning task**	**I-MG**	14.69 ± 10.12	28
One-on-one extended discussion with one or a few individuals	I-1o1	2.01 ± 4.14	11
Showing a demo, experiment, simulation, video or animation	I-DV	3.71 ± 4.53	21
Administration	I-Adm	6.79 ± 3.56	31
Waiting	I-W	3.03 ± 4.29	18
Other	I-O	3.15 ± 3.49	21

Codes that begin with S or I are ‘students doing’ and ‘instructor doing’ codes, respectively. All definitions are from [12], with the exception of student group work (S- GW). Mean percentage of 2-minute intervals were calculated using means per class section. Activities in bold were retained for further analysis.

### Student performance

Because our data were based on different concept inventories across a variety of courses, we used a meta-analytic approach such that different outcomes could be standardized prior to comparison. Each of the 31 sections that we observed was considered a single ‘study’, and thus each section was treated as an independent data point in our analyses. To assess changes in student performance between the pre- and post-tests, we calculated an effect size for each section. We recognize inherent differences among concept inventories required given that the diversity of classes that were used in this study may have an effect on comparisons among classes; the standardization to effect sizes was done to dampen this effect, but it cannot remove it entirely. The effect size of the difference between pre- and post-test scores and its standard error within each class section were calculated using the standardized mean gain following Lipsey and Wilson [20]. Equations and full descriptions of these calculations can be found in the S3 File.

### Statistical analyses

Each course section was used as datum in our analyses, resulting in 31 individual data points. In all cases, the dependent variable in our analyses was the effect size for the standardized mean gain in a particular course section. We compared sets of generalized linear models to assess how well different predictors of student performance fit the observed patterns in learning gains. We used Akaike Information Criteria, corrected for sample size (AICc), to rank the models. A particular model was considered the single ‘best’ model if it had the lowest AICc value, and differed from the next best model by a value of 2 or more. All analyses were carried out in R 3.4.4 [21].

We first examined the relationship between the broad categories for ‘Instructional style’, outlined in Lund et al. [14] and the standardized mean gain within each course section. While these are very broad categorizations of classroom activity, we have included this analysis to examine the general trends in approaches to teaching. To add more detail to this approach, we then determined which class activities were the best predictors of learning gains, and assessed the effect of the duration of the seven COPUS variables outlined above on effect sizes. The three-way and all possible two-way interactions among S-GW, I-FUp, and I-MG were included to account for the potential interactive effects of instructor and student activities that occur during implementation of active learning approaches. All 304 possible models given the seven independent variables and specified interactions were compared using the *dredge* function in the MuMin package in R [22]. Predictor variables were standardized prior to model comparison. Because there was more than one ‘best’ model, we used a model averaging approach to extract regression coefficients from the top 2AICc models to determine the magnitude and direction of the most consistent predictors of learning gains [23]. As a part of this approach, we tested for collinearity between additive independent predictors by examining the variance inflation factors (vif), an indication of the severity of collinearity between predictor variables. Because I-Lec had a vif value larger than five and could influence the outcome of model averaging to yield spurious results [24], we ran the analyses with and without this variable included and they yielded the same results (i.e., I-Lec was dropped from the best models when included). Finally, we used the same generalized linear modeling framework to identify whether particular types of group work are more effective than others by comparing learning gains between course sections that used these approaches to those that did not. In this case, we compared two models for each type of group work: one that included presence/absence of the activity as a predictor of learning gains, and one that included only an intercept (i.e., a non-zero effect size with no influence of the activity on learning gains).

## Results

### Instructional styles and student performance

**Fig 1:**
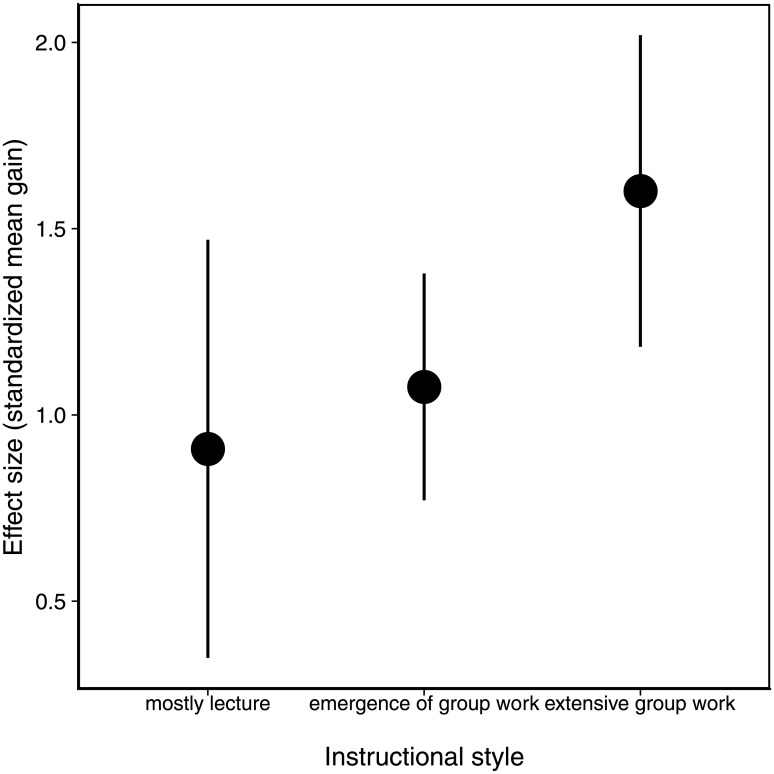
Student performance on concept diagnostics across classrooms using different instructional styles. Effect size is calculated as standardized learning gains. Error bars are 95% confidence intervals of the estimated effect sizes.

Of the 31 course sections observed here, five were classified as “Mostly Lecture,” 17 as “Emergence of Group Work,” and nine as “Extensive Group Work”. For all classroom profiles, effect sizes were positive, indicating that student performance improved between the pre- and post-test for all categories (Fig 1). However, the effect of instructional style on student learning gains was relatively weak; comparison of the model containing instructional style as a predictor and one that did not (an ‘intercept only’ model) revealed that these two models were equivalent (ΔAICc = 0.18). This result reflects the large variance within instructional styles, as despite the fact that the mean effect size for “Extensive Group Work” was 1.8 times higher than “Mostly Lecture”, the 95% confidence intervals for these values overlap (Fig 1).

### COPUS categories and student performance

**Fig 2:**
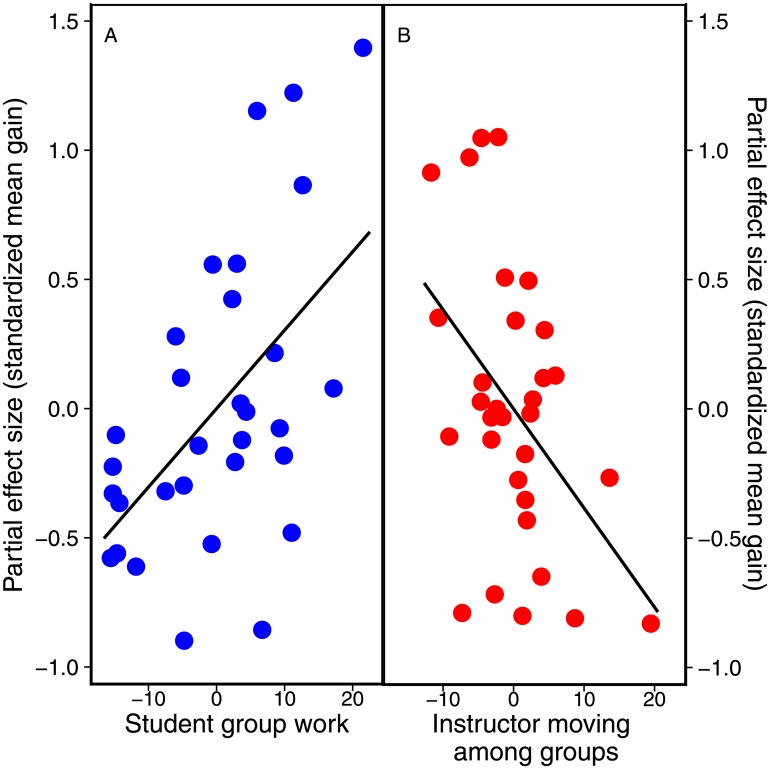
Student performance on concept diagnostics for COPUS variables that were the best predictors of learning gain. Effect sizes are standardized partial estimates holding all other variables constant to account for the influence of other predictors in the regressions for individual parameters; COPUS variables are standardized to account for differences in means across the independent variables.

The frequency and occurrence of all COPUS categories are shown in Table 2. Of the seven variables used in our analysis, six were retained in the top models: S-GW, S-AnQ, S-Q, I-RtW, I-FUp and I-MG (Table 3). Both S-GW and I-MG were retained in all five of the top 2AICc models, and their coefficients differed from zero for the averaged model as well as in each individual model (Table 3). These parameters influenced learning gains in opposite directions; S-GW had a positive relationship with effect size, while the effect of I-MG was negative (Table 3; Fig 2). Coefficients for the other four variables (S-AnQ, S-Q, I-RtW and I-FUp) were not consistently different from zero in the averaged model (Table 3). Thus, we did not consider these four variables as reliable predictors of learning gains.

**Table 3:** Summary of the top 2AICc models and the averaged model out of a possible 304 models assessing the influence of seven COPUS variables on student learning gains.

	Model parameter							
Model	S-GW	I-MG	I-RtW	S-AnQ	S-Q	I-FUp	R^2^	ΔAICc
1	**1.11 [0.53,1.69]**	**-0.92 [-1.55, -0.30]**	0.42 [-0.05,0.88]				0.37	0
2	**0.98 [0.39, .56]**	**-0.68 [-1.27, -0.10]**					0.30	0.77
3	**1.01 [0.44,1.57]**	**-0.74 [-1.36, -0.12]**	0.43[-0.02,0.88]	**-0.60 [-1.19,-0.02]**	0.53 [-0.05,1.12]		0.47	1.09
4	**1.07 [0.48,1.65]**	**-0.87 [-1.50, -0.24]**	0.41 [-0.06,0.87]	-0.22 [-0.64,0.21]			0.40	1.82
5	**1.22 [0.59,1.85]**	**-0.91 [-1.54, -0.29]**	**0.50 [0.002,1.01]**			-0.26 [-0.79,0.28]	0.40	1.96
**Averaged**	**1.07 [0.46,1.67]**	**-0.83 [-1.47,-0.18]**	0.33 [-0.21,0.88]	-0.14 [-0.69,0.41]	0.1 [-0.38,0.59]	-0.03 [-0.28,0.22]	0.45	

Values associated with each model parameter are the coefficients and 95% confidence intervals; if no value is given, the parameter was not retained in that model. Bold numbers have coefficients with confidence intervals that do not overlap zero, and are thus considered significant. R^2^ indicates the proportion of variance in effect size that is explained by the variance in the retained parameters, ΔAICc is the difference between a given model and the best model.

### Types of group work and student performance

**Fig 3:**
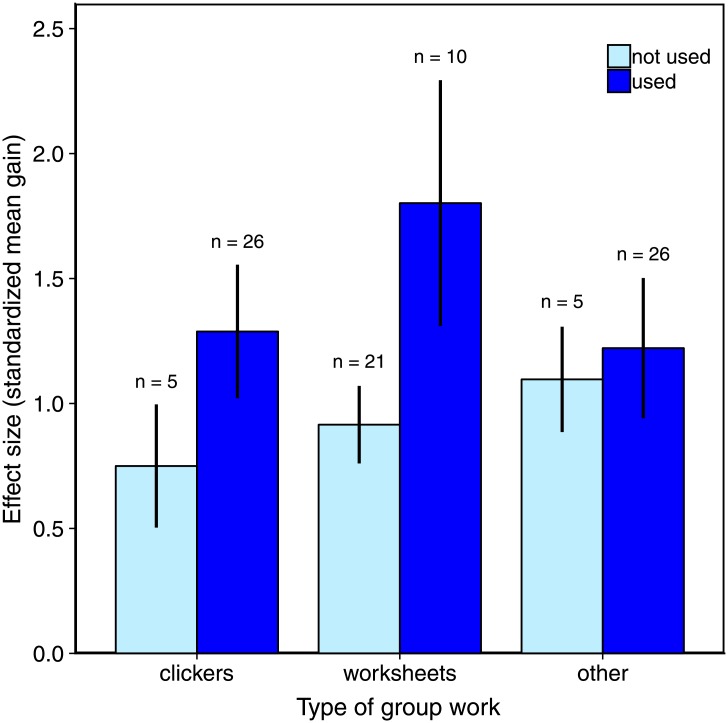
Effect sizes for learning gains on diagnostic tests comparing sections that used or did not use each type of group work. Error bars are 95% confidence intervals of effect size and sample sizes above bars indicate the number of sections.

To further examine the positive effect of group work on student learning, we split this variable into its component parts: i<Clicker questions for which students discuss their answers, worksheets, and ‘other types’ of activities that primarily involved an instructor showing a slide or overhead with a question and having the students answer it together in groups. We compared student performance between sections in which a particular activity occurred or not. Most sections had clicker questions (*n* = 26) and ‘other types’ of group work (*n* = 26); however, fewer than half of the observed sections used worksheets (*n* = 10; Table 2). Of the three types of group work, only the presence of worksheets had a clear effect on student performance when compared to an “intercept only” model ignoring worksheets (ΔAICc = 12.91; Fig 3). i<Clicker questions had a very weak effect on learning gains (ΔAICc = 0.44; Fig 3).

## Discussion

By combining direct, non-interventional classroom observations with quantitative assessments of learning gains across the Biology curriculum at a large university, we confirm the well-established positive effects of active learning on student conceptual understanding (e.g., [4,6]). Strikingly, we found that using in-class worksheets, a simple intervention with a low barrier to entry, resulted in significant increases in student scores. Thus, by using observations of classroom practices in conjunction with course-specific concept diagnostics, we were able to specify which types of student-centred activities support and promote conceptual learning.

### Student learning and classroom structure

Student performance was higher in classes that were characterized as consisting of Extensive Group Work, compared to the other two instructional styles (Mostly Lecture, Emergence of Group Work), although there was considerable variation within each category. The Extensive Group Work category is largely defined by class periods in which the instructor lectures for only half of the allotted time or less, while the majority of classroom activity involves student group work and follow-up discussions mediated by the instructor [14]. By contrast, the ‘Emergence of Group Work’ category features more than half of the class time spent on lecture, and less than 25% of the time on group work [14]. Our result that the highest learning gains occurred in classes with a considerable amount of group work is consistent with findings from Prather et al. [4], where classes that spent 25% of time or more on student centred teaching practices tended to have the highest learning gains. In addition, a recent investigation of the influence of moderate versus high use of student-centered classroom approaches in Introductory Biology indicated that an extensive amount of student activity, driven mainly by a difference in the frequency of group work, improved student performance and attitudes about the topic [6]. Our results, coupled with other studies, suggest that class time investment in group work will result in higher learning gains.

While a broad categorization of instructional styles allows for a general characterization of the classroom, COPUS data on specific student and instructor actions can provide greater resolution regarding the types of activities that may be most beneficial for conceptual understanding. Indeed, given the uncertainty surrounding the comparison among instructional styles, the best positive predictor of student performance was the time allocated to student group work. Group work is often used to typify situations that would be considered ‘active learning’, and several meta-analyses have indicated that active learning practices in general enhance student learning [3,4,7]. In addition, collaborative classrooms dedicate a large portion of time-on-task to student discussion, which allows for engagement with the course material through explanation and discussion that can maximize student learning [13,25,26]. In our averaged model, a 10% increase in group work time (five minutes in a 50-minute class) correlates with a 0.30 increase in effect size—roughly a 3% improvement in student performance (almost one letter grade, depending on the institution), holding other variables constant. In a broader perspective, simply giving five minutes of time for group work has similar impact as the use of a “researcher-developed” or “specialized” intervention [27]. Further highlighting the potential impact of such a low-barrier intervention, studies with effect sizes greater than 0.20 are noted to be of interest in educational policy decisions [7]. Our study is particularly relevant in this context, as current policy looks towards more diversity and inclusion in STEM, and active-learning approaches are known to close the achievement gap for students from disadvantaged backgrounds and minorities [2,7,28].

Surprisingly, our best models indicated that “Instructors moving in groups” (I-MG) was a negative predictor of student learning. In our study, the “instructor” included both teaching assistants and the lecturer. There are several reasons why class time spent on discussions with individual groups might negatively affect learning. First, the nature of these interactions, and impact on the group(s) directly participating, are unknown; our broad-scale observational approach does not capture the nuances of the interactions between instructor and student groups. For example, during short discussions, the nature of student-instructor interactions has been shown to alter the quality of student interactions, particularly if reasoning is provided by the instructor rather than allowing the students to express their rationale [29,30]. In addition, in large-enrollment classes such as the majority of those assessed here, instructors are only able to interact with a relatively small number of the groups within the classroom; this is further influenced by the tiered layout of large classes, such that instructors do not always have the ability to access the entire room effectively. This has the potential to reduce the engagement level of students in groups that are not targeted, which might reduce learning gains for the class as a whole. Further directed study to address interactions between instructors and groups is needed to verify and resolve this negative effect.

The four other variables that were retained in the best models had much lower explanatory power. Two of these variables, Student Asks a Question (S-Q) and Instructor Follow-up (I-FUp), were retained in only one model and did not have coefficients that differed from zero. Both instructor real-time writing (I-RtW) and student answering a question posed by the instructor (S-AnQ) were retained in more than one of the best models; however for each variable the confidence intervals around the estimated coefficients did not overlap zero for only one model, and both had confidence intervals that overlapped zero in the averaged model. This suggests that their influence on student learning is not strong compared to the other predictors on the models. The amount of time spent on real-time-writing by the instructor (I-RtW) was a weakly positive indicator of student learning; this may be attributed to the fact that real time writing was often observed during follow-up after a group activity. Additionally, real-time writing may result in a decrease in the pace of the classroom allowing students more time to synthesize information and take additional hand-written notes, a behavior that increases gains on conceptual questions compared to using a laptop ([31], but see [32] for a replicate study reporting a non-significant effect). By contrast, student answering a question in front of the whole class (S-AnQ) was a weakly negative predictor in our models. This effect may be attributed to a decrease in student engagement during this activity, particularly in large, acoustically-poor lecture halls. Students may disengage if the discussion between the individual student and the instructor lasts too long; indeed, there may be an optimal duration of this activity that allows all students to remain engaged, or, there may be other instructional approaches to avoid this issue entirely while still eliciting student responses (e.g., having Teaching/Learning Assistants summarize student responses to the whole class). Data on these aspects of classroom interactions are lacking.

### Components of group work: Worksheets and peer instruction

Time spent on group work emerged as an important predictor of learning gains and therefore we further investigated any possible effects of specific sub-types of group work. Strikingly, worksheets had a strong effect on increasing student performance, despite the variability in worksheet styles and practices implemented across courses. This finding promotes a simple and accessible method to support student learning. Worksheets do not necessarily require large time investments by the instructor for development or feedback; their construction can be relatively straightforward such as using questions based on previous tests or problem sets, and they need not be handed in for grading. Furthermore, they do not require special technology for implementation or student engagement. The benefit of even the simplest of worksheets is that they encourage students to articulate, evaluate, and reflect on a written answer. The simultaneous or sequential combination of peer discussion and writing has been shown to enhance student understanding and retention of complex concepts in STEM education [33–35], and this type of effect may explain the learning gains associated with worksheets in our study. Furthermore, this type of activity has been implemented in STEM classrooms [36–39], and assessment of student learning indicates that worksheets increase conceptual understanding when used as part of an active learning curriculum [36,39]. In the courses that we observed, many of the worksheets were guided activities that were embedded within the lectures or were ‘case-study’ approaches that required students to examine multiple aspects of a particular problem. While the use of worksheets resulted in higher gains compared to student response systems in our study, most of the courses we assessed used that technology, limiting our ability to detect an effect. The effectiveness of student response systems for student learning has been documented previously (e.g., [39,40]), and the adoption of this tool at our institution was widespread for this reason.

### Variation in the impact of active learning approaches

While classroom practices accounted for approximately 45% of the variability in student performance, our approach did not capture several important aspects of student learning. First, this study examined only in-class activities. Students spend a non-trivial amount of time on class preparation, homework, and studying. From a national survey, this university’s student population has a self-reported average of 19 study hours weekly [41]; this value includes students from all faculties and is likely an underestimate for STEM students [42]. Further, individual student characteristics and perceptions impact their practices and experiences, even within an active-learning classroom [2,43–45]. Even in classes where the amount of assigned work is equivalent, time-on-task outside the classroom can vary across students and may be affected by various factors relating to course structure, allotment of grades, and instructor characteristics. Studies that further investigate these questions should include the amount, and the type, of work that students undertake outside of class, as well as instructor expectations of their work. Second, the COPUS observational tool only captures the amount of time spent in a particular classroom practice. It does not distinguish between different implementations of the same practice, which can significantly impact the effectiveness of any classroom approach [46]. This can include student accountability (such as whether or not participation marks are allocated, or if clicker questions are graded, or if worksheets are handed in [36,47]), content-specific and content-independent instructor cues during peer instruction [2,13,48,49], and Bloom’s level and scaffolding of the worksheet/clicker questions [37]. Third, our study did not target comparisons between courses in upper and lower years, and thus did not capture differences in learning as students move through the curriculum and mature as learners. It is important to note that in this study the courses with the highest effect sizes were from the first and second years of the program. Finally, our analysis does not take into account temporal spacing in a classroom, such as the order of content, practice, feedback, the length of particular group work sessions, or the distinction between individual and group work. These variables are likely to be very important for student learning, as seen in other studies [50–53]. Tools that allow for the analysis and flow-of-time visualization of COPUS data are sorely needed to support research that will investigate the impact of how class time evolves on student outcomes.

### Implications for teaching

Given the large number of variables that can impact student learning, it is indeed notable that student performance increased with the simple inclusion of more group work. The finding that student performance can be predicted by in-class time underscores the importance of the structure and use of in-class time to facilitate achievement of learning goals. The frequent calls for changes to STEM education cannot, and should not, be ignored; however, the process of changing one’s approach to using class time is not trivial. Our results indicate that even a relatively short duration of group work can lead to increases in student learning. As an instructional tool, we would suggest that educators consider the use of structured worksheets as a way to increase student-centered use of class-time. Using this easy-to-implement, low-technology teaching practice will encourage collaboration, problem solving, and can be used to inform the instructor about what students are struggling with, providing opportunities for valuable and timely feedback.

## Supporting information

S1 File Conceptual inventory scores for all matched pre- and post-tests. (XLSX)

S2 File COPUS observation data. Section numbers correspond to the sections outlined in S1 File.
(XLSX)

S3 File Supplementary methods. (PDF)

S1 Table Summary of concept inventory test sizes and question sources. (PDF)

S2 Table Correlation matrix for all COPUS categories. Values above the diagonal are correlation coefficients; bold values are significant at the p<0.05 level. Values below the diagonal are p-values. Red fill indicates significant negative correlations; green fill denotes significant positive correlations.
(PDF)

S1 Fig Mean discrimination indices of test questions. **A.** Questions are categorized based on source (homemade were designed by authors and instructors; mod. validated were previously published/validated questions that were modified slightly; and validated were questions that were published and validated by the source authors). **B**. Questions are categorized based on source and course level (upper vs. lower division). Error bars are standard error of the mean.
(PDF)

## References

[1] Deslauriers L, Schelew E, Wieman C. Improved learning in a large-enrollment physics class. Science. 2011;332: 862–864. 10.1126/science.1201783 21566198

[2] Eddy SL, Hogan KA. Getting under the hood: how and for whom does increasing course structure work? CBE Life Sci Educ. 2014;13: 453–468. 10.1187/cbe.14-03-0050 25185229 PMC4152207

[3] Hake RR. Interactive-engagement versus traditional methods: A six-thousand-student survey of mechanics test data for introductory physics courses. Am J Phys. 1998;66: 64–74. 10.1119/1.18809

[4] Prather EE, Rudolph AL, Brissenden G, Schlingman WM. A national study assessing the teaching and learning of introductory astronomy. Part I. The effect of interactive instruction. Am J Phys. 2009;77: 320–330. 10.1119/1.3065023

[5] Freeman S, O’Connor E, Parks JW, Cunningham M, Hurley D, Haak D, et al Prescribed active learning increases performance in introductory biology. CBE Life Sci Educ. 2007;6: 132–139. 10.1187/cbe.06-09-0194 17548875 PMC1885904

[6] Connell GL, Donovan DA, Chambers TG. Increasing the use of student-centered pedagogies from moderate to high improves student learning and attitudes about biology. CBE Life Sci Educ. 2016;15: 1–15. 10.1187/cbe.15-03-0062 26865643 PMC4803092

[7] Freeman S, Eddy SL, McDonough M, Smith MK, Okoroafor N, Jordt H, et al Active learning increases student performance in science, engineering, and mathematics. Proc Natl Acad Sci U S A. 2014;111: 8410–8415. 10.1073/pnas.1319030111 24821756 PMC4060654

[8] Lund TJ, Stains M. The importance of context: an exploration of factors influencing the adoption of student-centered teaching among chemistry, biology, and physics faculty. Int J STEM Educ. 2015;2: 13 10.1186/s40594-015-0026-8

[9] Ebert-May D, Derting T, Hodder J, Momsen J, Long T, Jardeleza S. What we say is not what we do: effective evaluation of faculty professional development programs. Bioscience. 2011;61: 550–558. 10.1525/bio.2011.61.7.9

[10] Sawada D, Piburn MD, Judson E, Turley J, Falconer K, Benford R, et al Measuring reform practices in science and mathematics classrooms: the reformed teaching observation protocol. Sch Sci Math. 2002;102: 245–253. 10.1111/j.1949-8594.2002.tb17883.x

[11] Wieman C, Gilbert S. The teaching practices inventory: a new tool for characterizing college and university teaching in mathematics and science. CBE Life Sci Educ. 2014;13: 552–569. 10.1187/cbe.14-02-0023 25185237 PMC4152215

[12] Smith MK, Jones FHM, Gilbert SL, Wieman CE. The classroom observation protocol for undergraduate STEM (COPUS): a new instrument to characterize university STEM classroom practices. CBE Life Sci Educ. 2013;12: 618–627. 10.1187/cbe.13-08-0154 24297289 PMC3846513

[13] Eddy SL, Converse M, Wenderoth MP. PORTAAL: a classroom observation tool assessing evidence-based teaching practices for active learning in large science, technology, engineering, and mathematics classes. CBE Life Sci Educ. 2015;14: 1–16. 10.1187/cbe-14-06-0095 26033871 PMC4477739

[14] Lund TJ, Pilarz M, Velasco JB, Chakraverty D, Rosploch K, Undersander M, et al The best of both worlds: building on the COPUS and RTOP observation protocols to easily and reliably measure various levels of reformed instructional practice. CBE Life Sci Educ. 2015;14: 1–12. 10.1187/cbe.14-10-0168 25976654 PMC4477734

[15] Adams WK, Wieman CE. Development and validation of instruments to measure learning of expert-like thinking. Int J Sci Educ. 2011;33: 1289–1312. 10.1080/09500693.2010.512369

[16] Smith JI, Tanner K. The problem of revealing how students think: concept inventories and beyond. CBE Life Sci Educ. 2010;9: 1–5. 10.1187/cbe.09-12-0094 20194800 PMC2830154

[17] Williams KS, Heinrichsen ET. Concept Inventories/Conceptual Assessments in Biology (CABs). In: San Diego State University Centre for Teaching and Learning [Internet]. 2018 [cited 30 Jul 2019]. https://go.sdsu.edu/dus/ctl/cabs.aspx

[18] Wolak ME, Fairbairn DJ, Paulsen YR. Guidelines for estimating repeatability. Methods Ecol Evol. 2012;3: 129–137.

[19] Cicchetti D V. Guidelines, criteria, and rules of thumb for evaluating normed and standardized assessment instruments in psychology. Psychol Assess. 1994;6: 284–290. 10.1037/1040-3590.6.4.284

[20] Lipsey MW, Wilson DB. Practical meta-analysis. Thousand Oaks, CA: Sage Publications; 2001.

[21] R Core Team. R: A language and environment for statistical computing. R Foundation for Statistical Computing, Vienna, Austria [Internet]. 2011 http://www.r-project.org/

[22] Barton K. MuMIn: Multi-Model Inference [Internet]. 2018. https://cran.r-project.org/package=MuMIn

[23] Burnham KP, Anderson DR. Model selection and multimodel inference: a practical information-theoretic approach. 2nd ed Berlin: Springer; 2002.

[24] Cade BS. Model averaging and muddled multimodel inferences. Ecology. 2015;96: 2370–2382. 26594695 10.1890/14-1639.1

[25] Willoughby T, Wood E, McDermott C, McLaren J. Enhancing learning through strategy instruction and group interaction: is active generation of elaborations critical? Appl Cogn Psychol. 2000;14: 19–30. 10.1002/(SICI)1099-0720(200001)14:1<19::AID-ACP619>3.0.CO;2-4

[26] Dunlosky J, Rawson KA, Marsh EJ, Nathan MJ, Willingham DT. Improving students’ learning with effective learning techniques: promising directions from cognitive and educational psychology. Psychol Sci Public Interes Suppl. 2013;14: 4–58. 10.1177/1529100612453266 26173288

[27] Lipsey MW, Puzio K, Yun C, Hebert MA, Steinka-Fry K, Cole MW, et al Translating the statistical representation of the effects of education interventions into more readily interpretable forms. (NCSER 2013–3000) Natl Cent Spec Educ Res Inst Educ Sci US Dep Educ. Washington, DC; 2012.

[28] Eddy SL, Brownell SE. Beneath the numbers: a review of gender disparities in undergraduate education across science, technology, engineering, and math disciplines. Phys Rev Phys Educ Res. 2016;12: 20 10.1103/PhysRevPhysEducRes.12.020106

[29] Knight JK, Wise SB, Rentsch J, Furtak EM. Cues matter: Learning assistants influence introductory biology student interactions during clicker-question discussions. CBE Life Sci Educ. 2015;14: 1–14. 10.1187/cbe.15-04-0093 26590204 PMC4710402

[30] Kulatunga U, Lewis JE. Exploration of peer leader verbal behaviors as they intervene with small groups in college general chemistry. Chem Educ Res Pract. 2013;14: 576–588. 10.1039/c3rp00081h

[31] Mueller PA, Oppenheimer DM. The pen is mightier than the keyboard: advantages of longhand over laptop note taking. Psychol Sci. 2014;25: 1159–1168. 10.1177/0956797614524581 24760141

[32] Morehead K, Dunlosky J, Rawson KA. How much mightier is the pen than the keyboard for note-taking? A replication and extension of Mueller and Oppenheimer (2014). Educ Psychol Rev. 2019;

[33] Rivard L, Straw SB. The effect of talk and writing on learning science: an exploratory study. Sci Educ. 2000;84: 566–593. 10.1002/1098-237X(200009)84:5<566::AID-SCE2>3.3.CO;2-L

[34] Cross DI. Creating optimal mathematics learning environments: combining argumentation and writing to enhance achievement. Int J Sci Math Educ. 2009;7: 905–930. 10.1007/s10763-008-9144-9

[35] Menekse M, Stump GS, Krause S, Chi MTH. Differentiated overt learning activities for effective instruction in engineering classrooms. J Eng Educ. 2013;102: 346–374. 10.1002/jee.20021

[36] Sujarittham T, Emarat N, Arayathanitkul K, Sharma MD, Johnston I, Tanamatayarat J. Developing specialized guided worksheets for active learning in physics lectures. Eur J Phys. 2016;37: 025701 10.1088/0143-0807/37/2/025701

[37] Leslie-Pelecky DL. Interactive worksheets in large introductory physics courses. Phys Teach. 2000;38: 165 10.1119/1.880485

[38] Bridgeman AJ. Using very short writing tasks to promote understanding in chemistry. Proc Aust Conf Sci Math Educ. 2012; 110–117.

[39] Meltzer DE, Manivannan K. Transforming the lecture-hall environment: the fully interactive physics lecture. Am J Phys. 2002;70: 639 10.1119/1.1463739

[40] Smith MK, Wood WB, Adams WK, Wieman C, Knight JK, Guild N, et al Why peer discussion improves student performance on in-class concept questions. Science. 2009;323: 122–124. 10.1126/science.1165919 19119232

[41] Schwartz Z. Where Students Study the Most 2016: Full Results. Maclean’s Magazine. Mar 2016. https://www.macleans.ca/education/where-students-study-the-most-full-results/

[42] National Survey of Student Engagement. Promoting Student Learning and Institutional Improvement: Lessons from NSSE at 13. Natl Surv Student Engagem. 2012; 50.

[43] Cooper KM, Brownell SE. Coming out in class: challenges and benefits of active learning in a biology classroom for LGBTQIA students. CBE Life Sci Educ. 2016;15: 1–19. 10.1187/cbe.16-01-0074 27543636 PMC5008884

[44] Cooper KM, Downing VR, Brownell SE. The influence of active learning practices on student anxiety in large-enrollment college science classrooms. Int J STEM Educ. International Journal of STEM Education; 2018;5: 23 10.1186/s40594-018-0123-6 30631713 PMC6310416

[45] Corkin DM, Horn C, Pattison D. The effects of an active learning intervention in biology on college students’ classroom motivational climate perceptions, motivation, and achievement. Educ Psychol. Routledge; 2017;37: 1106–1124. 10.1080/01443410.2017.1324128

[46] Borrego M, Cutler S, Prince M, Henderson C, Froyd JE. Fidelity of implementation of research-based instructional strategies (RBIS) in engineering science courses. J Eng Educ. 2013;102: 394–425. 10.1002/jee.20020

[47] Polodak K, Danforth J. Interactive modern physics worksheets methodology and assessment. Eur J Phys Educ. 2013;4: 27–31.

[48] Lewin JD, Vinson EL, Stetzer MKR, Smith MK. A campus-wide investigation of clicker implementation: the status of peer discussion in STEM classes. CBE Life Sci Educ. 2016;15: 1–12. 10.1187/cbe.15-10-0224 26931397 PMC4803095

[49] Caldwell JE. Clickers in the large classroom: current research and best-practice tips. CBE Life Sci Educ. 2007;6: 9–20. 10.1187/cbe.06-12-0205 17339389 PMC1810212

[50] Bruff D. Teaching with classroom response systems: creating active learning environments. John Wiley and Sons; 2009.

[51] Crouch CH, Mazur E. Peer instruction: ten years of experience and results. Am J Phys. 2001;69: 970–977. 10.1119/1.1374249

[52] Schwartz DL, Bransford JD. A time for telling. Cogn Instr. 1998;16: 367–398.

[53] Smith MK, Wood WB, Krauter K, Knight JK. Combining peer discussion with instructor explanation increases student learning from in-class concept questions. CBE Life Sci Educ. 2011;10: 55–63. 10.1187/cbe.10-08-0101 21364100 PMC3046888

