# Supplementary material for: Small changes, big gains: A curriculum-wide study of teaching practices and student learning in undergraduate biology
Source: PLoS One. 2019 Aug 28;14(8):e0220900. doi: 10.1371/journal.pone.0220900 (PMC6713325; doi:10.1371/journal.pone.0220900)
Supplement: S3 File — (PDF) [file pone.0220900.s003.pdf]

Small changes, big gains: a curriculum-wide study of teaching practices and student learning in undergraduate biology

Laura K. Weir[1#a]*, Megan K. Barker[1,#b], Lisa M. McDonnell[1,#c], Natalie G. Schimpf[1,#d], Tamara M. Rodela[1,#e] and Patricia M. Schulte[1,#d]

[1]Carl Wieman Science Education Initiative, University of British Columbia, Vancouver, British Columbia, Canada

[#a]Department of Biology, Saint Mary's University, Halifax, Nova Scotia, Canada

[#b]Department of Biological Sciences, Simon Fraser University, Burnaby, British Columbia, Canada

[#c]Section of Cell and Developmental Biology, University of California San Diego, La Jolla, California, United States of America

[#d]Department of Zoology, University of British Columbia, Vancouver, British Columbia, Canada

[#e]Department of Biology, St. Francis Xavier University, Antigonish, Nova Scotia, Canada

*Corresponding author

E-mail:laura.weir@smu.ca (LW)

Supplementary methods

Diagnostic test analysis

Discrimination index values were calculated for each question using post-test scores, using the following formula:

$$\frac{(\#students\ correct\ within\ upper\ group) - (\#\ students\ correct\ within\ lower\ group)}{(\#\ students\ per\ group)}$$

Upper and lower groups were the top and bottom 27% of the section population [1], based on their entire post-test score. For each test administered, a mean discrimination index was calculated from the discrimination indexes of each question. A discrimination index value can range from -1 to +1, with values higher than +0.2 considered fair to good [2]. The mean discrimination index of all tests ranged from 0.32 to 0.55 (overall mean for all tests 0.42 $\pm$ 0.22 (SD), median 0.41). Discrimination index summary and analysis results can be found in Table S1 and Fig S1.

Effect size calculations

The effect size of the difference between pre- and post-test scores within each class section was calculated using the standardized mean gain according to Lipsey and Wilson [3]. The equation for this effect size (ES) metric is:

$$ES = \frac{\bar{G}}{s_g / \sqrt{2(1-r)}}$$

where $\bar{G}$ is the mean post-test minus pre-test gain score within a given section, s_g is the standard deviation of the gain scores, and r is the correlation between pre-test and post-test scores.

References

1. Kelley TL. The selection of upper and lower groups for the validation of test items. J Educ Psychol. 1939;30: 17–24.

2. Ebel RL, Frisbie DA. Essentials of educational measurement. Englewood Cliffs, NJ: Prentice-Hall; 1986.

3. Lipsey MW, Wilson DB. Practical Meta-Analysis. Thousand Oaks, CA: Sage Publications; 2001.
