# Supplementary material for: Small changes, big gains: A curriculum-wide study of teaching practices and student learning in undergraduate biology
Source: PLoS One. 2019 Aug 28;14(8):e0220900. doi: 10.1371/journal.pone.0220900 (PMC6713325; doi:10.1371/journal.pone.0220900)
Supplement: S1 Table — (PDF) [file pone.0220900.s004.pdf]

S1 Table. Summary of concept inventory test sizes and question sources.

Course Level	Number of Students	Lower or Upper Division	Total No. of Questions	No. Home-made questions	No. Validated Questions	No. Modified Validated Questions	Question sources
100	1160	L	8	1	6	1	1
100	1900	L	23	4	3	16	2-7
200	1209	L	9	4	4	1	1, 8, 9
200	551	L	10	6	3	1	1, 10, 11
200	497	L	14	3	6	5	2, 6, 12
200	277	L	17	7	4	6	8, 10, 13-15
300	120	U	8	0	8	0	7
300	238	U	10	4	6	0	16,17
300	219	U	12	5	5	2	2,11,12
300	227	U	6	6	0	0	none
300	73	U	8	5	2	1	9, 11
400	19	U	12	4	6	1	11
400	12	U	8	5	2	1	9, 11
400	35	U	20	11	5	4	2, 6, 11, 12

References

1. Shi J, Wood W, Martin J, Guild N, Vicens Q, Knight JK. A diagnostic assessment for introductory molecular biology. Cell Biol Educ. 2010;9: 453–461. doi:10.1187/cbe.10
2. Smith MK, Wood WB, Knight JK. The Genetics Concept Assessment: a new concept inventory for gauging student understanding of genetics. CBE Life Sci Educ. 2008;7: 422–430. doi:10.1187/cbe.08
3. Abraham JK, Perez KE, Price RM. The dominance concept inventory: A tool for assessing undergraduate student alternative conceptions about dominance in mendelian and population genetics. CBE Life Sci Educ. 2014;13: 349–358. doi:10.1187/cbe.13-08-0160
4. Price RM, Andrews TC, McElhinny TL, Mead LS, Abraham JK, Thanukos A, et al. The genetic drift inventory: A tool formeasuring what advanced undergraduates have mastered about genetic drift. CBE Life Sci Educ. 2014;13: 65–75. doi:10.1187/cbe.13-08-0159
5. Baum DA. The Tree-Thinking Challenge. Science. 2005;310: 979–980. doi:10.1126/science.1117727
6. Kalas P, O’Neill A, Pollock C, Birol G. Development of a meiosis concept inventory. CBE Life Sci Educ. 2013;12: 655–664. doi:10.1187/cbe.12-10-0174

7. Hansen M. Population Dynamics Concept Inventory [Internet]. 2013. Available: http://q4b.biology.ubc.ca/concept-inventories/population-dynamics/
8. Klymkowsky MW, Underwood SM, Garvin-Doxas RK. Biological Concepts Instrument (BCI): A diagnostic tool for revealing student thinking. arXiv:10124501. 2010; doi:abs/1012.4501
9. Deane T, Nomme K, Jeffery E, Pollock C, Birol G. Development of the biological experimental design concept inventory (BEDCI). CBE Life Sci Educ. 2014;13: 540–551. doi:10.1187/cbe.13-11-0218
10. Maskiewicz AC, Griscom HP, Welch NT. Using targeted active-learning exercises and diagnostic question clusters to improve students' understanding of carbon cycling in ecosystems. CBE Life Sci Educ.
11. Couch BA, Wood WB, Knight JK. The molecular biology capstone assessment: A concept assessment for upper-division molecular biology students. CBE Life Sci Educ. 2015;14: 1–11. doi:10.1187/cbe.14-04-0071
12. Taylor J, Oh-McGinnis R, Chowrira S, Smith K. Transcription and Translation Concept Inventory [Internet]. 2013. Available: http://q4b.biology.ubc.ca/concept-inventories/transcription-and-translation/
13. Michael JA, Wenderoth MP, Modell HI, Cliff W, Horwitz B, McHale P, et al. Undergraduates' understanding of cardiovascular phenomena. Adv Physiol Educ. 2002;26: 72–84. doi:10.1152/advan.00002.2002
14. Michael JA, Richardson D, Rovick A, Modell H, Bruce D, Horwitz B, et al. Undergraduate students' misconceptions about respiratory physiology. Adv Physiol Educ. 1999;22: S127–S135. Available: http://advan.physiology.org/content/277/6/S127
15. Fisher KM, Williams KS, Lineback JE. Osmosis and diffusion conceptual assessment. CBE Life Sci Educ. 2011;10: 418–429. doi:10.1187/cbe.11-04-0038
16. Knudson D, Noffal G, Bauer J, McGinnis P, Bird M, Chow J, et al. Development and evaluation of a biomechanics concept inventory. Sports Biomech. 2003;2: 267–77. doi:10.1080/14763140308522823
17. Martin J, Mitchell J, Newell T. Development of a concept inventory for fluid mechanics. 33rd Annu Front Educ 2003 FIE 2003. 2003;1: 23–28. doi:10.1109/FIE.2003.1263340
