# Supplementary material for: Small changes, big gains: A curriculum-wide study of teaching practices and student learning in undergraduate biology
Source: PLoS One. 2019 Aug 28;14(8):e0220900. doi: 10.1371/journal.pone.0220900 (PMC6713325; doi:10.1371/journal.pone.0220900)
Supplement: S2 Table — Values above the diagonal are correlation coefficients; bold values are significant at the p<0.05 level. Values below the diagonal are p-values. Red fill indicates significant negative correlations; green fill denotes significant positive correlations. (PDF) [file pone.0220900.s005.pdf]

S2 Table. Correlation matrix for all COPUS categories.

	SL	Sind	SCG	SWG	SOG	SGW	SANQ	SQ	SWC	SPrd	SP	SW	SO	Ilec	IRtW	IFUp	IPQ	ICQ	IAnQ	IMG	I1o1	IDV	Iadm	IW	IO	
**SL**		-0.03	0.04	**-0.58**	**-0.53**	**-0.66**	0.05	0.24	-0.12	0.06	-0.10	-0.08	**-0.38**	**0.76**	-0.22	-0.17	0.01	0.01	0.21	**-0.77**	**-0.67**	**0.50**	0.18	-0.08	**-0.61**	**SL**
**Sind**	0.89		0.24	0.15	0.09	0.21	0.10	-0.02	0.01	0.27	-0.13	-0.09	0.23	-0.23	0.14	0.27	0.21	**0.39**	-0.03	0.07	-0.25	0.08	0.23	-0.19	0.31	**Sind**
**SCG**	0.82	0.20		0.08	-0.08	**0.58**	-0.07	0.04	-0.12	-0.18	-0.22	0.25	-0.08	0.02	-0.26	0.24	0.09	**0.95**	0.17	-0.09	-0.02	-0.06	0.19	**0.50**	-0.08	**SCG**
**SWG**	<0.001	0.42	0.67		-0.08	**0.49**	-0.13	-0.11	0.15	-0.11	0.09	-0.20	0.35	**-0.42**	0.17	0.19	-0.02	0.18	-0.03	**0.36**	0.17	**-0.36**	-0.17	-0.12	0.24	**SWG**
**SOG**	<0.01	0.62	0.67	0.68		**0.56**	0.17	-0.08	0.26	0.32	0.27	0.03	-0.06	**-0.63**	0.18	**0.42**	0.23	-0.07	-0.12	**0.79**	**0.44**	-0.33	-0.02	0.15	0.33	**SOG**
**SGW**	<0.001	0.27	<0.001	<0.01	<0.01		-0.03	-0.07	0.15	0.05	0.09	0.13	0.12	**-0.65**	0.06	**0.50**	0.15	**0.60**	0.03	**0.66**	**0.41**	**-0.45**	0.02	**0.40**	0.34	**SGW**
**SANQ**	0.81	0.61	0.71	0.50	0.37	0.86		**0.70**	0.09	-0.11	-0.01	-0.09	-0.08	-0.24	0.03	**0.54**	**0.92**	-0.12	**0.66**	0.10	-0.06	-0.20	0.23	-0.10	-0.08	**SANQ**
**SQ**	0.19	0.90	0.82	0.55	0.68	0.69	<0.001		0.22	-0.13	0.04	-0.08	-0.18	0.03	-0.10	0.31	**0.61**	-0.03	**0.96**	-0.11	-0.14	-0.17	0.24	-0.18	-0.31	**SQ**
**SWC**	0.51	0.96	0.53	0.43	0.16	0.43	0.65	0.24		-0.06	**0.91**	-0.13	-0.01	-0.15	-0.01	0.03	0.04	-0.18	0.11	**0.38**	-0.04	-0.10	-0.20	-0.16	-0.22	**SWC**
**SPrd**	0.73	0.14	0.33	0.55	0.08	0.78	0.57	0.50	0.74		-0.03	-0.11	-0.13	-0.20	0.08	0.26	-0.14	-0.06	-0.15	0.07	-0.09	0.06	-0.01	-0.02	-0.03	**SPrd**
**SP**	0.58	0.50	0.23	0.64	0.14	0.63	0.97	0.84	<0.001	0.86		-0.11	-0.04	-0.20	0.05	0.04	-0.05	-0.25	-0.09	**0.42**	-0.03	-0.15	-0.28	-0.13	-0.17	**SP**
**SW**	0.66	0.62	0.17	0.28	0.86	0.48	0.63	0.68	0.48	0.57	0.57		0.25	-0.18	0.15	0.10	-0.13	0.29	-0.04	0.02	0.07	-0.13	0.19	**0.46**	0.31	**SW**
**SO**	0.04	0.22	0.67	0.06	0.74	0.52	0.67	0.34	0.95	0.47	0.82	0.18		-0.28	0.32	-0.08	-0.03	0.06	-0.14	0.09	-0.03	-0.13	0.01	0.05	**0.60**	**SO**
**Ilec**	<0.001	0.22	0.90	0.02	<0.01	<0.001	0.19	0.86	0.41	0.27	0.29	0.34	0.12		**-0.47**	**-0.69**	-0.26	-0.09	0.02	**-0.83**	-0.27	**0.58**	0.08	-0.02	**-0.49**	**Ilec**
**IRtW**	0.23	0.44	0.16	0.37	0.32	0.74	0.88	0.60	0.97	0.68	0.77	0.42	0.08	<0.01		**0.39**	0.06	-0.17	-0.13	**0.36**	-0.16	-0.19	-0.20	-0.11	0.27	**IRtW**
**IFUp**	0.35	0.14	0.20	0.32	0.02	<0.01	<0.01	0.09	0.85	0.15	0.82	0.58	0.69	<0.001	0.03		**0.56**	0.32	0.31	**0.47**	-0.12	**-0.44**	0.09	0.12	0.07	**IFUp**
**IPQ**	0.98	0.26	0.63	0.90	0.22	0.42	<0.001	<0.001	0.81	0.45	0.78	0.48	0.88	0.16	0.74	<0.001		0.05	**0.62**	0.17	-0.06	-0.22	0.24	-0.03	0.00	**IPQ**
**ICQ**	0.97	0.03	<0.001	0.34	0.72	<0.001	0.53	0.89	0.34	0.74	0.17	0.11	0.76	0.64	0.37	0.08	0.78		0.10	-0.05	-0.11	-0.13	0.21	**0.42**	0.06	**ICQ**
**IAnQ**	0.26	0.87	0.36	0.85	0.54	0.89	<0.001	<0.001	0.57	0.42	0.63	0.85	0.45	0.90	0.50	0.09	<0.01	0.61		-0.13	-0.13	-0.16	0.27	-0.10	-0.30	**IAnQ**
**IMG**	<0.001	0.72	0.62	0.05	<0.001	<0.001	0.59	0.57	0.03	0.72	0.02	0.92	0.62	<0.001	0.05	<0.001	0.35	0.80	0.50		**0.43**	**-0.52**	-0.20	-0.04	**0.38**	**IMG**
**I1o1**	<0.001	0.17	0.92	0.35	0.01	0.02	0.73	0.46	0.81	0.63	0.88	0.69	0.87	0.15	0.39	0.53	0.75	0.55	0.48	0.02		-0.29	0.02	0.34	**0.42**	**I1o1**
**IDV**	<0.01	0.66	0.74	0.05	0.07	0.01	0.29	0.37	0.58	0.74	0.41	0.48	0.48	<0.001	0.31	0.01	0.23	0.49	0.40	<0.01	0.11		0.09	-0.11	-0.25	**IDV**
**Iadm**	0.34	0.21	0.30	0.36	0.93	0.91	0.22	0.20	0.29	0.96	0.12	0.29	0.96	0.65	0.27	0.63	0.19	0.26	0.14	0.29	0.93	0.63		0.22	0.01	**Iadm**
**IW**	0.68	0.30	<0.01	0.53	0.41	0.03	0.59	0.33	0.38	0.93	0.48	<0.01	0.81	0.92	0.54	0.51	0.86	0.02	0.58	0.81	0.06	0.57	0.24		0.20	**IW**
**IO**	<0.001	0.09	0.66	0.19	0.07	0.06	0.68	0.09	0.23	0.89	0.37	0.09	<0.001	<0.01	0.15	0.72	0.99	0.74	0.11	0.04	0.02	0.18	0.96	0.29		**IO**
	**SL**	**Sind**	**SCG**	**SWG**	**SOG**	**SGW**	**SANQ**	**SQ**	**SWC**	**SPrd**	**SP**	**SW**	**SO**	**Ilec**	**IRtW**	**IFUp**	**IPQ**	**ICQ**	**IAnQ**	**IMG**	**I1o1**	**IDV**	**Iadm**	**IW**	**IO**	

Values above the diagonal are correlation coefficients; bold values are significant at the p<0.05 level. Values below the diagonal are *p*-values. Red fill indicates significant negative correlations; green fill denotes significant positive correlations.
