# Supplementary figures and images for: Small changes, big gains: A curriculum-wide study of teaching practices and student learning in undergraduate biology
Source: PLoS One. 2019 Aug 28;14(8):e0220900. doi: 10.1371/journal.pone.0220900 (PMC6713325; doi:10.1371/journal.pone.0220900)

**Figure S1**

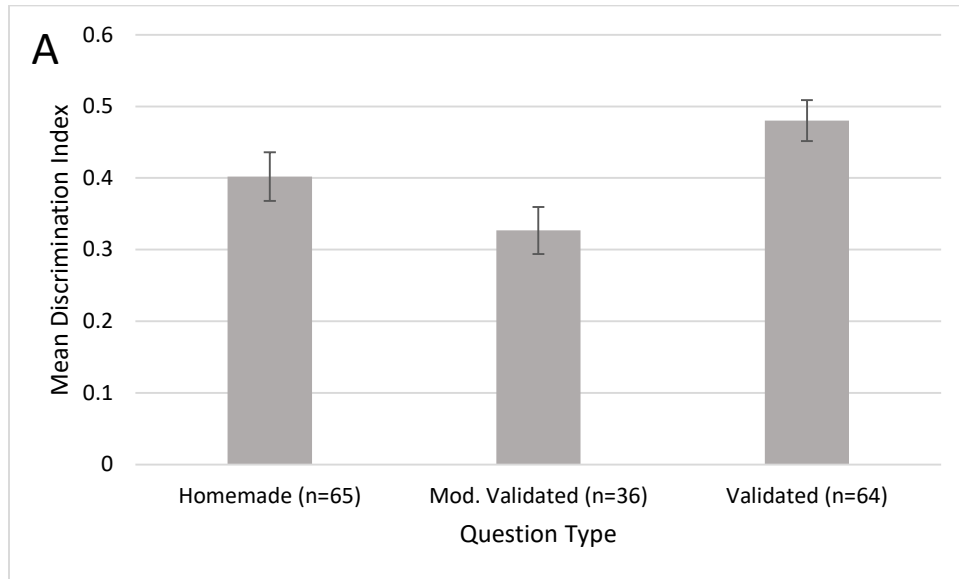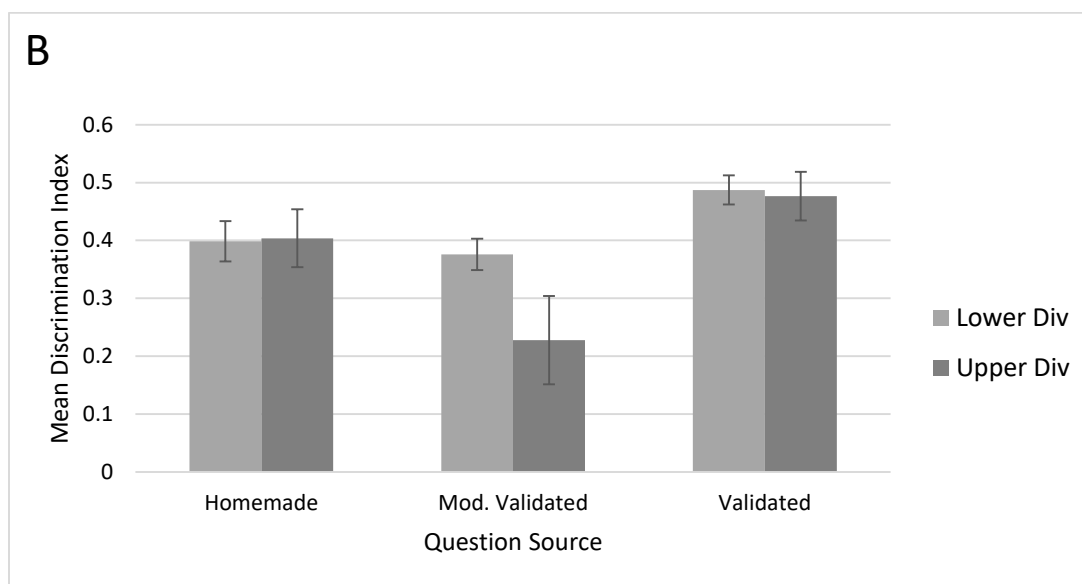

Supplement: S1 Fig — A. Questions are categorized based on source (homemade were designed by authors and instructors; mod. validated were previously published/validated questions that were modified slightly; and validated were questions that were published and validated by the source authors). B. Questions are categorized based on source and course level (upper vs. lower division). Error bars are standard error of the mean. (PDF) [file pone.0220900.s006.pdf]
